# Lactate Dehydrogenase-5 and Tumor-Infiltrating Lymphocytes in Prostate Cancer Patients Undergoing Radical Hypofractionated Radiotherapy

**DOI:** 10.3390/cancers18132149

**Published:** 2026-07-03

**Authors:** Ioannis M. Koukourakis, Kalliopi Platoni, Vassilis Kouloulias, Christina Yfanti, Stella Arelaki, Christos Kalaitzis, Anna Zygogianni, Michael I. Koukourakis, Alexandra Giatromanolaki

**Affiliations:** 1 Department of Radiotherapy/Oncology, School of Medicine, Democritus University of Thrace, 68100 Alexandroupolis, Greece; 2 Department of Pathology, School of Medicine, Democritus University of Thrace, 68100 Alexandroupolis, Greece; cyfanti@med.duth.gr (C.Y.); sarelaki@med.duth.gr (S.A.); agiatrom@med.duth.gr (A.G.); 3 Medical Physics Unit, 2nd Department of Radiology, School of Medicine, National and Kapodistrian University of Athens, Attikon University Hospital, 12462 Athens, Greece; kplatoni@med.uoa.gr; 4 Department of Clinical Radiation Oncology, Medical School, National and Kapodistrian University of Athens, Attikon University Hospital, 12462 Athens, Greece; vkouloul@med.uoa.gr (V.K.); azygogianni@med.uoa.gr (A.Z.); 5 Department of Urology, Democritus University of Thrace, 68100 Alexandroupolis, Greece; ckalaitz@med.duth.gr

**Keywords:** prostate cancer, radiotherapy, LDH, anaerobic metabolism, tumor-infiltrating lymphocytes

## Abstract

**Background/Objectives**: Aerobic glycolysis is a standard mechanism that cancer cells use to support their anabolic processes. Lactate and proton production, byproducts of this LDHA-catalyzed transformation of pyruvate, may contribute to acidifying the tumor microenvironment and to repressing the cytotoxic activity of anti-tumor immune cells. Prostate cancer radiotherapy outcome could be diminished by cancer cell metabolism and immunosuppression. **Methods**: The tumor infiltrating lymphocyte (TIL) density was assessed in 110 prostate adenocarcinoma biopsies from patients treated with radical radiotherapy. Immunohistochemistry was performed to evaluate LDH5 expression by cancer cells (encoded by the LDHA gene). **Results**: Higher tumor stages (T3 and T4) were associated with lower TIL density (*p* = 0.02). Approximately half of the patients (51%) displayed strong LDH5 cancer cell expression, which was linked with advanced T-stage and higher Gleason scores (*p* = 0.05 and *p* = 0.01, respectively). High LDH5 expression was significantly correlated with low TIL density (*p* ≤ 0.0004). In univariate analysis, high LDH5 and low TIL density were associated with poor biochemical relapse-free survival (BRFS) (*p* ≤ 0.006). In multivariate analysis, TIL density, but not LDH5, was an independent predictor of BRFS (*p* = 0.03). **Conclusions**: LDH5 overexpression is associated with low tumor lymphocytic infiltration. Stratifying prostate carcinomas according to LDH5/TIL density identifies a group of patients with low TIL density and high LDH5 expression, who have a higher risk of tumor relapse. Targeting glycolysis to disrupt cancer cell metabolism offers an interesting research area for prostate cancer therapy.

## Simple Summary

Pyruvate conversion to lactate for ATP production, catalyzed by LDH5, may hinder anti-tumor immunity, potentially through acidification of the tumor stroma, and may affect the outcome of radiotherapy. In biopsies from 110 prostate cancer patients, we assessed LDH5 expression by immunohistochemistry in cancer cells and tumor-infiltrating lymphocyte (TIL) density in H&E tissue sections. We identified a group of patients at a higher risk of biochemical relapse who displayed high LDH5 expression and low TIL density. Targeting glycolysis as a form of metabolic interference presents an interesting area for further research in prostate cancer treatment.

## 1. Introduction

Anaerobic usage of glucose for energy production even under well-oxygenated conditions is a hallmark of cancer cells, as first reported by Warburg et al. [1]. This so-called ‘aerobic glycolysis’ refers to the overactivation of ATP production through the transformation of pyruvate to lactate by the enzymatic activity of lactate dehydrogenase-alpha (LDHA)-composed isoenzymes of lactate dehydrogenase (LDH). Lactate dehydrogenase-5 (LDH5), composed of four M (muscle) subunits (M4) encoded by the LDHA gene, is the most potent enzyme catalyzing this reaction, while isoenzymes containing H (heart) subunits encoded by the LDHB gene catalyze the inverse reaction [2]. Although pyruvate entry into the Krebs’ cycle produces more ATP, bypassing oxidative phosphorylation through LDH provides very rapid ATP production, while also producing high levels of intermediate molecules to support anabolic reactions [3].

The LDH5 isoenzyme is highly expressed in all human carcinomas and overexpressed in a significant proportion of tumors, and can be assessed using immunohistochemistry with LDH5-specific antibodies [4]. Along with the metabolic advantage provided by LDH5 that promotes cancer cell proliferation and tumor aggressiveness, acidification of the tumor microenvironment through lactate and proton release provides an unparalleled pathway for tumors to escape immune surveillance. T-cell and NK cell proliferation and anti-tumor activity are significantly compromised under acidic conditions [5,6]. Moreover, histone lactylation—a consequence of lactate production—promotes overexpression of immunosuppressive immune-checkpoint molecules like PD-L1 [7].

Tumor-infiltrating lymphocyte (TIL) assessment is gradually evolving into an important biomarker to characterize an immunosuppressive tumor microenvironment, linked with poor prognosis in many human carcinomas [8]. Immunologically cold tumors, poorly infiltrated by lymphocytes, are resistant to novel immunotherapy with immune checkpoint inhibitors [9]. In addition, the efficacy of radiotherapy (RT) is also compromised in tumors with reduced immunological response [10,11]. Immunity-mediated tumor clearance has been recognized as an important mechanism of post-radiation tumor eradication [12].

Radical RT is a standard treatment option for patients with non-metastatic prostate cancer, with a 10-year survival ranging from 75–90% depending on risk factors [13,14]. Although Gleason score and T-stage are strongly linked to biochemical relapse after RT, additional molecular parameters, including cancer cell metabolism and immunity, may also influence RT efficacy, among a variety of others that shape the complex landscape of prostate cancer biology. In the current study, we investigated the role of LDH5 and tumor-infiltrating lymphocytes in prostate cancer and in the efficacy of RT.

## 2. Materials and Methods

Paraffin-embedded material from biopsies of 129 cancer patients with acinar prostate adenocarcinoma (non-metastatic to organs or regional lymph nodes) was obtained from the archives of the Department of Pathology, University Hospital of Alexandroupolis, Democritus University of Thrace. Our department manages a significant number of prostate cancer patients referred from different hospitals and centers throughout Northern Greece. The patients analyzed here are consecutive patients whose biopsies were performed only at our hospital (between 2007 and 2021), and for whom tissue material was available for the study. All patients received radical RT (without surgery), and those without biochemical relapse had at least five years of follow-up. We used the Phoenix definition of biochemical relapse, defined as a rise in Prostate-Specific Antigen (PSA) of ≥2 ng/mL above the nadir PSA level achieved after RT with or without androgen deprivation therapy (ADT) [15]. Thirty-three (30%) patients developed biochemical relapse during the follow-up period.

Initial staging included a digital rectal examination; prostate biopsy; multiparametric MRI assessment of the prostate and pelvis; CT evaluation of the pelvis, upper abdomen and chest; and bone scan scintigraphy. Eight patients with documented lymph node and/or distant organ metastasis (including oligometastatic disease) were excluded from the analysis. For five patients, tissue material was deemed inadequate because it had been exhausted for diagnostic or research purposes. Six patients were lost to follow-up before completing the minimum 5-year follow-up and were excluded from analysis. A total of 110 patients were included in the current analysis. The median follow-up of all patients after RT until biochemical relapse was 60 months (range, 6–180 months). For patients without biochemical relapse, the median follow-up was 72 months (range, 60–180 months). Details on patient age, tumor characteristics, and PSA serum levels are shown in Table 1.

All included patients provided written informed consent authorizing the anonymous use of their clinical and laboratory data for scientific research and publications. Obtaining written informed consent is a standard procedure used in our university department. All patients undergoing therapy are asked to provide this written informed consent, but declining does not affect any aspect of their therapy. The current manuscript presents results from a larger retrospective study examining the role of metabolism and immunity in the outcome of radical RT. It received approval from the Ethics and Research Committee of the University Hospital of Alexandroupolis (Approval number ES 20, 17 July 2025).

Patients with low/favorable intermediate-risk characteristics (44 patients) received RT to the prostate and seminal vesicles, without ADT. Patients with unfavorable intermediate or high/very high-risk tumors (66 patients) received RT to the prostate, seminal vesicles, and pelvic lymph nodes, with ADT for 18 months. The risk groups shown in Table 1 were defined according to the European Association of Urology Guidelines [16]. Briefly, this risk-scoring system is as follows: Low risk (Gleason 6 and PSA < 10 ng/mL and cT1,2); favorable intermediate (Gleason 3+4 and cT1,2 and PSA < 10 ng/mL or Gleason 6 and cT1,2 and PSA 10–20 ng/mL); unfavorable intermediate (Gleason 3+4 and PSA 10–20 ng/mL and cT1,2 or Gleason 4+3 and cT1,2 and PSA < 20 ng/mL); and high risk (Gleason 9–10 or PSA > 20 ng/mL or cT3,4 or lymph node involvement). RT was delivered using a hypofractionated and accelerated approach, administering 3.8 Gy to the prostate, 3.5 Gy to the seminal vesicles, and 2.75 Gy to the pelvic lymph nodes over 14 consecutive fractions within 18 days. A 3D (77 patients treated before 2018) or VMAT technique (33 patients treated after 2018) was used. Radiobiological analysis of these hypofractionated and accelerated RT schedules, showing superior biological dose delivered to the tumor while reducing the dose to the organs at risk, has been previously reported [17,18].

### 2.1. Assessment of TIL Density

Assessment of TIL density was performed on hematoxylin and eosin tissue sections. For each patient, 2–6 tissue blocks (median, 4) were available, each containing 2–8 biopsy cylinders/cores (median, 5). Expert pathologists examined the Hematoxylin and Eosin (H&E) tissue sections from all tissue blocks and identified one or two slides per patient containing the most suitable cancer tissue cores, ensuring that at least 8 cores would be available for assessment and immunohistochemistry.

Following evaluation of all available cores (8–15 per patient), four optical fields (o.f.) at ×200 magnification with the highest lymphocytic infiltration were selected, and the number of lymphocytes was counted, yielding four scores per biopsy. The mean and maximum TIL densities (TILmean and TILmax) were used to score each tumor for further analysis. TIL density was treated as a continuous variable in the analysis. For group analysis, the 25th and 33rd percentiles were used to split cases into two groups: low vs. medium–high TIL density. These cutoff points have been used by our group in studies examining the role of TIL density in non-small cell and breast cancers, showing that low TIL density is associated with poor prognosis [19,20].

Assessment was performed by two independent observers (AG, CY), and discrepancies were resolved using a conference microscope under the supervision of a senior pathologist (AG). Pathologists were blinded to all patient data, including other immunohistochemical marker analyses.

### 2.2. Assessment of LDH5 Expression

Details on LDH5 immunohistochemistry in prostate cancer have been previously reported by our group [21]. In this earlier study, LDH5 overexpression was linked to poor post-RT prognosis, while cell line experiments showed a direct association of LDH5 overexpression with radioresistance. Given the recent developments in immunotherapy, we assessed LDH5 in a new cohort of patients and examined its association with lymphocytic infiltration in prostate cancer, aiming to provide a better understanding of the complex interplay between anaerobic metabolism and immunity, beyond radioresistance.

Briefly, 3 μm thick sections containing prostate cancer cores (selected as mentioned above) were deparaffinized and placed in antigen retrieval target solution at pH 9.0 (Dako, Glostrup, Denmark), then microwaved (3 × 5 min). Non-specific binding was blocked by incubating with normal rabbit serum at a 1:20 dilution for 30 min (Dako, X0902, Glostrup, Denmark). The sheep polyclonal antibody to LDH-5 isoenzyme (ab 9002, Abcam, Cambridge, UK) was used at a 1:200 dilution for an overnight incubation at 4 °C. Sections were incubated with a secondary antibody (Biotinylated Secondary rabbit antisheep; Dako, P0163, Glostrup, Denmark) at a 1:100 dilution for 30 min. Streptavidin-HRP (K0679, Dako, Glostrup, Denmark) was applied for 30 min, and the color was developed by incubation with DAB solution. Then, sections were lightly counterstained with hematoxylin. Normal immunoglobulin G was used as a negative control instead of the primary antibody.

**Figure 1:**
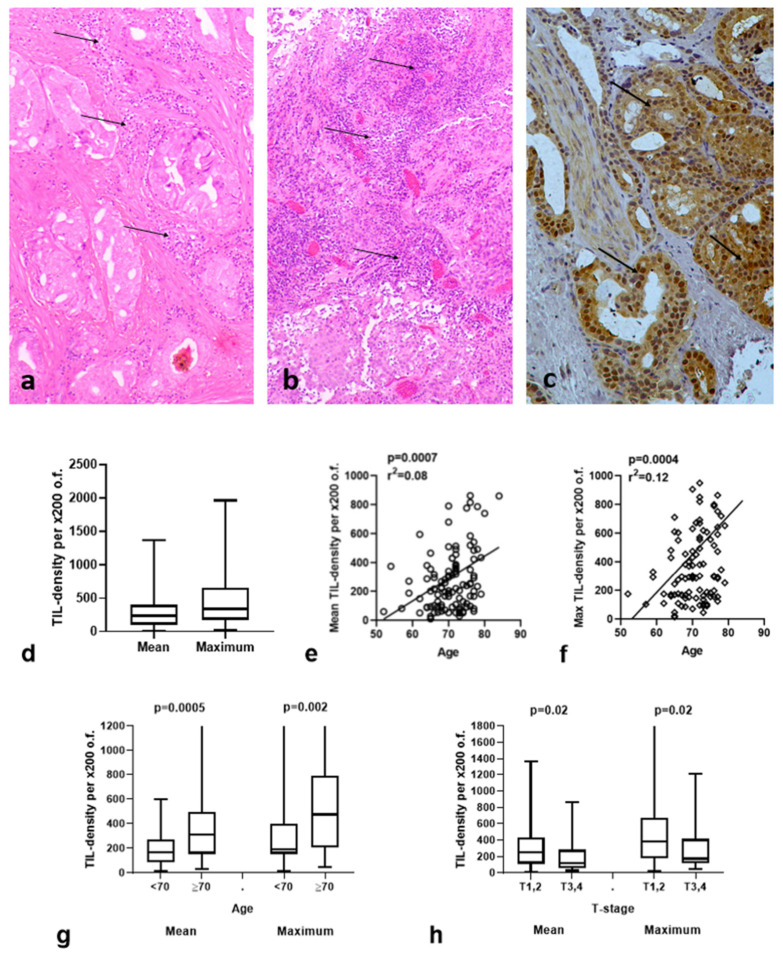
Typical pathology images of prostate carcinomas: (**a**) low lymphocytic infiltration (arrows; ×100), (**b**) high lymphocytic infiltration (arrows; ×100) and (**c**) strong nuclear and cytoplasmic expression of LDH5 (arrows; ×200). Mean and maximum tumor-infiltrating lymphocyte densities per ×200 optical field (o.f.) (TILmean and TILmax (**d**); their association with age by linear regression analysis (**e**,**f**); group analysis (**g**) and T-stage (**h**). Box and whiskers show minimum, maximum, and median values, as well as the 25th and 75th percentiles.

The nuclear and the strong cytoplasmic expression were separately evaluated in all available ×200 o.f. by determining the percentage of cancer cells expressing LDH5 (Figure 1c). The average of these percentages was used to score each case. Using a previously established scoring system that takes into consideration both cytoplasmic and nuclear expression [22], tumors were classified into two groups: low and high LDH5 expression. Patients with strong cytoplasmic LDH5 expression in more than 50% of cancer cells and/or nuclear expression in more than 10% of cancer cells were classified as having high LDH5 expression.

### 2.3. Statistical Analysis

Statistical analysis was conducted using GraphPad 8.1 and IBM SPSS v25 software. Comparison of continuous variables between two groups was performed with the unpaired non-parametric Mann–Whitney test. Fisher’s exact test was used to compare categorical variables. Categorical TIL analysis was performed using the 25th and 33rd percentiles. Time-dependent ROC analysis was also conducted in relation to biochemical relapse. Biochemical relapse-free survival (BRFS) was estimated with Kaplan–Meier curves, and comparisons were performed using the log-rank test. Despite the overall low number of cases, a Cox proportional hazards model with backward conditional elimination, including variables significant in univariate analysis, was used to evaluate independence. TIL density was used as both the dichotomous and continuous variables in multivariate models. A two-tailed *p*-value of less than 0.05 was regarded as statistically significant.

## 3. Results

Figure 1a,b show typical images of prostate carcinomas with low and high TIL densities. The nuclear and strong cytoplasmic expression of LDH5 is shown in Figure 1c.

### 3.1. TIL Density and Correlation with Tumor Parameters

The TILmean ranged from 11 to 1370 lymphocytes per ×200 o.f. (median, 232). The maximum TIL density (TILmax) ranged from 15 to 1962 (median, 338) (Figure 1d). We used the 25th and 33rd percentiles of TIL density values to define two groups as low vs. medium/high TIL density. For TILmean, the values were 95 and 190, respectively. For TILmax, these were 170 and 151, respectively. Using the 25th percentile, 30/110 patients and 29/110 patients had low TILmean and TILmax, respectively. Using the 33rd percentile, 37/110 patients had low TILmean and 37/110 had low TILmax, respectively.

In linear regression analysis, a direct correlation was observed between high TILmean and TILmax with advanced age (*p* = 0.0007, r = 0.31, r^2^ = 0.08 and *p* = 0.0004, r = 0.33, r^2^ = 0.12, respectively); the group analysis in Figure 1e,f revealed significantly lower TILmean and TILmax in patients younger than 70 years old (Figure 1g). The median TILmean and TILmax were 169 and 189, respectively, in younger patients, compared to 310 and 475 in older patients (*p* < 0.001).

Regarding T-stage, T3 and T4 tumors were associated with significantly lower TILmean and TILmax (Figure 1h). The median TILmean value was 114 vs. 253 in T3,4 and T1,2 cases, respectively (*p* = 0.02). The median TILmax was 173 vs. 383, respectively (*p* = 0.02).

No association between TIL densities and Gleason score or serum PSA levels was noted.

### 3.2. LDH5 Expression and Correlation with Tumor Parameters

The median strong cytoplasmic expression was 30% (range, 0–80%) and the median nuclear expression was 5% (range, 0–60%). Using a previously developed scoring system by our group (see Materials and Methods), 56/110 (51%) patients were considered to have high LDH5 expression. High LDH5 expression was significantly linked with advanced T-stage (*p* = 0.05) and high Gleason score (*p* = 0.01). In Table 2, no association was found with PSA levels (*p* = 0.34) or patient age (*p* = 0.22). A strong association of high LDH5 expression with low TIL density (mean and maximum) was noted (*p* < 0.0001 and 0.0004, respectively). When TIL density was used as a continuous variable, cases with high LDH5 showed median TILmean and TILmax values of 130 and 186, respectively. In contrast, cases with low LDH5 had TILmean and TILmax values of 292 and 465, respectively. These differences between LDH5 groups are statistically significant (*p* = 0.0007 and 0.0004).

### 3.3. Biochemical Relapse-Free Survival

**Figure 2:**
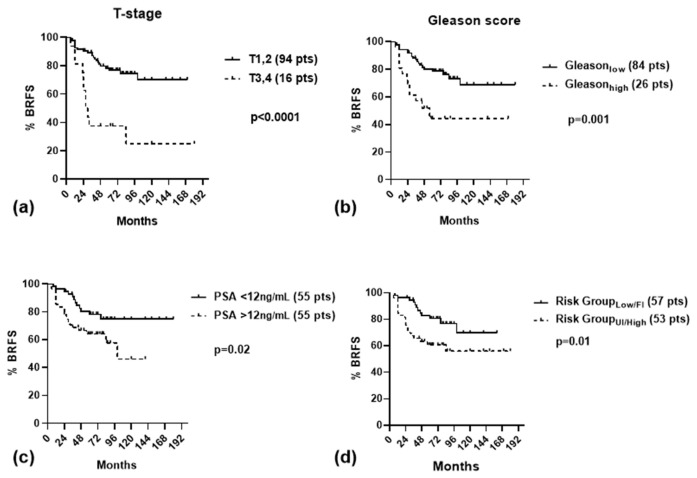
Kaplan–Meier biochemical relapse-free survival (BRFS) after radical radiotherapy according to T-stage (**a**), Gleason-score (**b**), PSA serum levels (**c**), and risk group of patients (**d**). FI = favorable intermediate, UI = unfavorable intermediate risk groups.

Kaplan–Meier analysis of BRFS showed a significant association of poor prognosis with advanced T-stage (T1,2 vs. T3,4; *p* < 0.0001), high Gleason score (6/3+4 vs. 4+3/8–10; *p* = 0.001), and PSA levels (lower vs. higher than the median 12 ng/mL; *p* = 0.001). In Figure 2a–c, although younger age (<70) was linked to worse outcomes, this did not reach significance (*p* = 0.16). The unfavorable intermediate/high-risk group of patients had a poorer BRFS (*p* = 0.01; Figure 2d).

**Figure 3:**
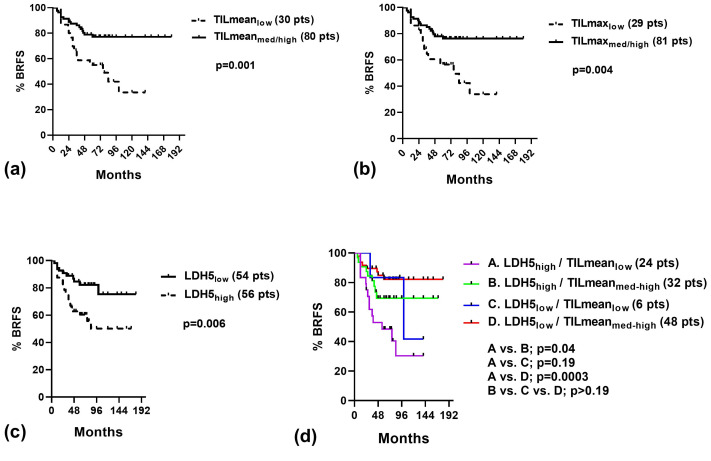
Kaplan–Meier biochemical relapse-free survival (BRFS) after radical radiotherapy according to mean tumor-infiltrating lymphocyte (TIL) density (TILmean) (**a**), maximum TIL density (TILmax) (**b**), LDH5 expression (**c**), and a double-stratification analysis of TILmean and LDH5 (**d**). TILmean groups are defined by the 25th percentile of TILmean values.

Time-dependent ROC analysis of TIL density against BRFS suggested that the best cutoff points at the 60-month landmark for TILmean and TILmax scores were 110 (AUC 0.55, 95% CI 0.41–0.69) and 170 (AUC 0.56, 95% CI 0.42–0.69), respectively, with no significant discriminative ability at this time-point. These values correspond to the 29th and 26th percentiles, respectively. Analysis, however, was performed using the 25th and 33rd percentiles, as described in the Materials and Methods. Both the 25th and 33rd TIL percentiles, used as cutoff points, defined groups of patients with significant differences in BRFS. The 25th cutoff point for TILmean yielded a *p*-value of 0.001 and a hazard ratio (log rank) of 2.80 (95% CI 1.27–6.16), while TILmax yielded a *p*-value of 0.004 and a hazard ratio of 2.58 (95% CI 1.69–5.73). The 33rd cutoff point for TILmean yielded a *p*-value of 0.01 and a hazard ratio of 2.23 (95% CI 1.07–4.63), while TILmax yielded a *p*-value of 0.01 and a hazard ratio of 1.072–4.61. Figure 3a,b show the Kaplan–Meier analyses of BRFS for TILmean and TILmax, respectively, with patients grouped by the 25th percentile of TIL values. The analysis based on LDH5 expression also showed a significant association of high expression with poor BRFS (*p* = 0.006). Figure 3c depicts a double-stratification analysis showing that tumors with high LDH5 and low TILmean were associated with the worst outcome (Figure 3d). This also applied to the TILmax/LDH5 analysis.

We further performed multivariate analyses using different prognostic models, employing backward conditional Cox regression analysis (Table 3). Because there were 33 events of biochemical relapse among 100 patients, a safe analysis allows for only three variables in the models. We therefore considered two models that included the ‘risk group’ as defined in the Materials and Methods (low/favorable intermediate vs. unfavorable intermediate/high), TILmean or TILmax groups (defined by the 25th percentile of TIL values), and LDH5 (low vs. high). TILmean and TILmax were independent prognostic indicators of BRFS, whereas LDH5 was not (*p*-value: 0.004 and 0.006, respectively). Taking into account the suggestion from the univariate analysis that cases with a high LDH5 and low TILmean or TILmax had the worst BFRS, we assessed a bivariate model including the risk grouping and the double-stratified LDH5/TIL score (high/low vs. all others). LDH5/TILmean and LDH5/TILmax were independent prognostic factors for BRFS (*p* = 004 and 0.007, respectively; Table 3).

Moreover, we performed a similar regression analysis including the three variables, but TILmean or TILmax were introduced as continuous variables. In these models, TIL was not a significant predictor of BRFS (TILmean: *p* = 0.71; TILmax: *p* = 0.33). However, the risk group and LDH5 remained independently associated with BRFS (when TILmean was introduced: *p* = 0.04 and *p* = 0.03, respectively; when TILmax was introduced: 0.04 and 0.04, respectively).

**Figure 4:**
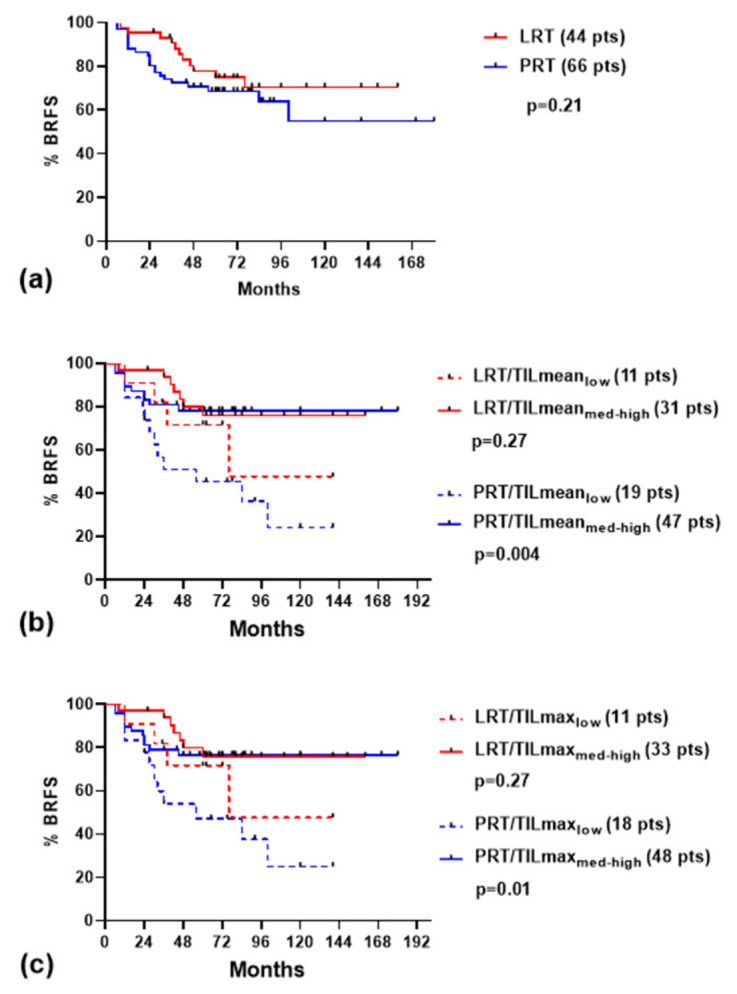
Kaplan–Meier biochemical relapse-free survival (BRFS) after radical radiotherapy according to: (**a**) type of radiotherapy (localized RT-LRT vs. pelvic RT and androgen deprivation therapy ADT–PRT/ADT); (**b**) double stratification by type of radiotherapy and mean tumor-infiltrating lymphocyte (TIL) density (TILmean); and (**c**) double stratification by type of radiotherapy and maximum TIL density (TILmax). TIL groups are defined by the 25th percentile of TIL values.

We further performed an exploratory analysis of the prognostic role of TILmean and TILmax (defined by the 25th percentile TIL value) in patients treated with localized RT to the prostate and seminal vesicles (LRT; 44 patients) and in patients who also received pelvic RT and ADT (PRT/ADT; 66 patients). This analysis, however, cannot predict the interaction between TILs and ADT or lymph node irradiation, because ADT was given only to patients receiving pelvic RT. Results should therefore be considered indicative. Although the group of patients receiving PRT/ADT showed a trend toward poorer BRFS, the difference did not reach significance (*p* = 0.21; Figure 4a). Low TILmean was associated with poorer BRFS in patients receiving PRT/ADT (*p* = 0.004), while this was not significant in the group of patients receiving LRT (*p* = 0.27). Figure 4b shows similar results in the TILmax analysis, where low TILmax was associated with poor BRFS in patients treated with PRT/ADT (*p* = 0.01), whereas this association did not reach significance in the group treated with LRT (*p* = 0.27; Figure 4c).

## 4. Discussion

Metabolism and immunity are interconnected molecular and physiological processes that influence cancer growth, survival, and resistance to treatments. Using LDH5, the key enzyme responsible for converting pyruvate to lactate for energy production—making it a central regulator of the Warburg effect—we analyzed its expression in a series of biopsies from prostate cancer patients treated with radical RT. In accordance with a previously published series from our group [21], LDH5 was highly expressed in 50% of cases. Although hypoxic upregulation of LDH5 via HIF1α stabilization and binding to the LDHA gene promoter may explain the common overexpression of LDH5 [23], suppressed LDHB gene activity due to hypermethylation of the LDHB promoter also appears to be a common event in prostate cancer, which promotes LDH5 overexpression [24]. Repression of the LDHB gene and activation of the LDHA gene may also occur due to growth factors released in the prostate tumor environment, such as fibroblast growth factor [25].

Overexpression of LDH5 was associated with an advanced T-stage, a high Gleason score, and poorer BRFS following radiation therapy. Although, to our knowledge, there are no other studies on tissue LDH expression in prostate cancer, an adverse prognostic role of serum LDH has been reported in patients with non-metastatic disease who had positive pelvic lymphadenopathy treated with RT [26]. Elevated serum LDH levels are linked to reduced overall survival in patients with metastatic prostate cancer [27]. Furthermore, high serum LDH levels have been associated with a reduced response to new antiandrogens, abiraterone, and also to cabazitaxel chemotherapy [28,29]. In light of recent advances in understanding androgen signaling biology and its link to glycolysis, these data could be useful in research aimed at overcoming resistance to hormonal therapy [30].

Although the present study does not provide direct mechanistic evidence that LDH5-overexpressing tumors release substantial amounts of lactate and protons into the tumor stroma, robust experimental data support a link between LDH5, lactate production, and matrix acidification [31,32]. This acidification is likely to hinder lymphocyte proliferation and activity within the tumor. Although the role of the anti-tumor immune response in prostate cancer is not well understood and immune checkpoint inhibitors have shown no clinical effect, adoptive immunotherapy with activated TILs, as reported in a case report, suggested significant antitumor activity [33]. Despite the generally accepted concept that prostate cancer is an immunologically ‘cold’ tumor, this might not always be the case [34]. In the current study, low TIL density was observed in only one-third of patients, while the rest had several hundred to over one thousand lymphocytes per ×200 o.f. in biopsy samples. The importance of TIL density was validated by its direct link to an advanced T-stage, high Gleason score, and its independent prognostic value in patients receiving radical RT. The nature of TIL subtypes may also be significant, as regulatory T-cells could be associated with poor outcomes [35,36]. In contrast, dense infiltration by cytotoxic CD8+ T-cells has been associated with improved survival in patients undergoing prostatectomy [37].

Histopathological correlations revealed that low TIL density was associated with younger patient age and advanced T-stage. It could be speculated that immunological impairment may promote early disease onset. A family history of prostate cancer increases the risk only at a younger age, and this may show a genetic dissociation across age [38]. In support of this finding, SPOP mutations, which are common in early-onset prostate cancer, have been linked to impaired ubiquitination of PD-L1 via the Cullin-3 E3 ubiquitin ligase complex, eventually reducing TIL density in the tumor microenvironment [39,40]. The above discussion should be viewed as a speculative effort to offer a plausible explanation, but it needs to be tested genetically in a series of patients with early onset of the disease or cohorts with confirmed hereditary prostate carcinomas. The association of low lymphocytic infiltration with advanced T-stage may imply a more rapid growth in the setting of impaired immune surveillance. This hypothesis is further supported by the direct correlation of low TIL density with LDH5 expression, which sustains glycolytic tumor metabolism and creates an unfavorable microenvironment for TILs. LDH5 was also linked to advanced T-stage and Gleason score. Concomitant LDH5 overexpression and low TIL density defined the subgroup of patients with the worst BRFS.

In multivariate analysis, TILmean and TILmax had independent prognostic relevance as dichotomous variables. However, this was not verified when these were considered as continuous variables. Using time-dependent ROC analysis, the suggested cutoff values at the 60-month landmark were between the 26th and 29th percentiles, closely matching the prespecified 25th percentile used for the original analysis (although the 33rd percentile yielded similar results), despite the AUC at this landmark not reaching statistical significance. The fact that TIL density lost its independent prognostic value when considered as a continuous variable may further indicate that the relationship between BRFS and TIL density is non-linear rather than absent. Nevertheless, these findings should be considered exploratory and should be validated within an independent cohort. Moreover, the observation that LDH5 loses its independent prognostic relevance once TIL parameters are included as dichotomous variables in the regression model may suggest the presence of additional LDH5-independent immunosuppressive pathways, e.g., toll-like receptor and adenosine receptor expression, expansion of myeloid cells, and others [41,42,43].

This is a retrospective analysis from a single institution, based on archived tissue material from a small sample size, and should be considered as a pilot study for future prospective trials. Although patients were treated sequentially, the sample analyzed included only those with tissue available at our hospital, so selection bias cannot be excluded. Lack of independent validation is also acknowledged. In addition, characterization of the lymphocytic component with specific markers that identify cytotoxic, regulatory, or exhausted T-cells would further contribute to the elucidation of the role of the immune response in prostate cancer, and is currently under investigation by our group. An additional limitation of the study is the assessment of TILs and LDH5 in pre-RT biopsies, whereas post-RT biopsies may provide better insights into adaptation of antitumor immunity and may have prognostic relevance. However, such studies require a prospective design because prostate biopsy after RT is not a routine procedure. The analysis presented here, despite the exploratory double stratification analysis (TILs vs. RT/ADT), cannot predict interactions between TILs and ADT or lymph node irradiation because pelvic irradiation and ADT were offered only to high-risk patients.

## 5. Conclusions

LDH5 overexpression is associated with a compromised immune response in prostate cancer, as indicated by the low tumor lymphocytic infiltration, which aligns with the expected acidosis prevailing in the tumor microenvironment. Stratifying prostate carcinomas according to LDH5/TIL density can be readily done using prostate cancer biopsy samples. This approach identifies a group of patients with low TIL density and high LDH5 expression (~25% of all those referred for RT) who have a higher risk of tumor relapse. This proposed combined TIL/LDH5 parameter should be considered as an exploratory biomarker for further validation. Whether these patients might gain from more intensive treatment, such as pelvic RT combined with novel antiandrogens (enzalutamide or apalutamide [44]) therapy and/or chemotherapy, remains to be explored through clinical research. Policies to inhibit LDH5 expression with HIF-inhibitors or LDHA blockers could be tested in this patient subgroup [45,46].

## Figures and Tables

**Table 1:** Patient and disease characteristics.

No Patients	110
Age	
Range (52–84)	
Median (71)	
<70	39
≥70	71
Histology	
Acinar adenocarcinoma	110
Gleason score	
6	62
3+4	22
4+3	8
8–10	18
Grade group	
1	62
2	22
3	8
4	10
5	8
T-stage (Clinical) (*)	
T1,2	94
T3	14
T4	2
N-stage (MRI)	
N0	110
PSA (ng/mL)	
<10 vs. ≥10 (**)	43 vs. 67
<12 vs. ≥12 (***)	55 vs. 55
<20 vs. ≥20 (**)	83 vs. 27
Risk group	
Low	33
Favorable intermediate	24
Unfavorable intermediate	10
High/Very high	43
RT technique	
Conformal	77
VMAT	33
RT fields	
Localized	44
Pelvic (****)	66
Biochemical relapse	
No	77
Yes	33

(*) Based on multiparametric MRI and biopsy. (**) Suggested PSA threshold values used in assessing risk groups. (***) PSA cutoff point corresponding to the median value in the current series of patients. (****) These patients also received ADT.

**Table 2:** Association of LDH5 expression with patient and disease parameters.

	LDH		*p*-Value
	Low	High	
Age *			**0.22**
<71	16 (14.6%)	23 (20.9%)	
≥71	38 (34.5%)	33 (30.0%)	
**T-stage**			**0.05**
1, 2	50 (45.5%)	44 (40%)	
3, 4	4 (3.6%)	12 (10.9%)	
**Gleason score**			**0.01**
6/3+4	47 (42.7%)	37 (33.6%)	
≥4+3	7(6.4%)	19 (17.3%)	
**PSA(1)**			**0.69**
<10 ng/mL	20 (18.1%)	23 (21.0%)	
≥10 ng/mL	34 (30.9%)	33 (30.0%)	
**PSA(2)**			**0.34**
<12 ng/mL	30 (27.3%)	25 (22.7%)	
≥12 ng/mL	24 (21.8%)	31 (28.2%)	
**PSA (1)**			**0.99**
<20 ng/mL	41 (37.3%)	42 (38.2%)	
≥20 ng/mL	13 (11.8%)	14 (12.7%)	
Risk group **			**0.13**
Low/FI	32 (29.1%)	25 (22.7%)	
UI/High	22 (20.0%)	31 (28.2%)	
TILmean ***			**0.0002**
Low	6 (5.5%)	24 (21.8%)	
Medium–high	48 (43.6%)	32 (29.1%)	
TILmax ***			**0.0004**
Low	6 (5.5%)	23 (20.9%)	
Medium–high	48 (43.6%)	33 (30.0%)	

* Age median value 71. ** FI = favorable intermediate, UI = unfavorable intermediate. *** Cutoff value defined by the 25th percentile of TIL values. (1) Suggested PSA threshold values used in assessing risk groups. (2) PSA cutoff point corresponding to the median value in the current series of patients.

**Table 3:** Backward conditional Cox regression analysis of variables predicting biochemical relapse-free survival in multivariate analysis.

	*p*-Value	Hazard Ratio	95% CI	
			Lower	Upper
**Model 1 (including risk group, LDH5 and TILmean)**				
Risk group (*) (UI/High vs. Low/FI)	0.02	2.25	1.10	4.59
TILmean (**) (low vs. high)	0.004	2.76	0.39	5.48
LDH5 (***) (high vs. low)	0.16	1.80	0.78	4.11
**Model 2 (including risk group, LDH5 and TILmax)**				
Risk group (*) (UI/High vs. Low/FI)	0.02	2.07	1.00	4.26
TILmax (**) (high vs. low)	0.006	2.60	1.30	5.17
LDH5 (***) (high vs. low)	0.12	1.88	0.83	4.26
**Bivariate Model 3 (including risk group, combined LDH5/TILmean)**				
Risk group (*) (UI/High vs. Low/FI)	0.06	2.00	0.97	4.11
LDH5/TILmean (High/Low vs. all others)	0.004	2.83	1.40	5.71
**Bivariate Model 4 (including Risk group and combined LDH5/TILmax)**				
Risk group (*) (UI/High vs. Low/FI)	0.04	2.06	1.00	4.23
LDH5/TILmax (High/Low vs. all others)	0.007	2.66	1.31	5.40

(*) UI = unfavorable intermediate risk group, FI = favorable intermediate risk group. (**) TILmean and TILmax: groups defined using the 25th percentile of TIL values as a cutoff point. (***) LDH5 values obtained when all three variables were included in the model.

## Data Availability

The data that support the findings of this study are available from the corresponding author, M.I.K., upon reasonable request.
